# PNU-74654 Induces Cell Cycle Arrest and Inhibits EMT Progression in Pancreatic Cancer

**DOI:** 10.3390/medicina59091531

**Published:** 2023-08-24

**Authors:** Tai-Long Chien, Yao-Cheng Wu, Hsiang-Lin Lee, Wen-Wei Sung, Chia-Ying Yu, Ya-Chuan Chang, Chun-Che Lin, Chi-Chih Wang, Ming-Chang Tsai

**Affiliations:** 1 Department of Gastroenterology, Antai Medical Care Corporation Antai Tian-Sheng Memorial Hospital, Pingtung 928, Taiwan; 2 School of Medicine, Chung Shan Medical University, Taichung 402, Taiwan; 3 Institute of Medicine, Chung Shan Medical University, Taichung 402, Taiwan; 4 Department of Surgery, Chung Shan Medical University Hospital, Taichung 402, Taiwan; 5 Department of Urology, Chung Shan Medical University Hospital, Taichung 402, Taiwan; 6 Division of Gastroenterology and Hepatology, Department of Internal Medicine, Chung Shan Medical University Hospital, Taichung 402, Taiwan

**Keywords:** pancreatic adenocarcinoma, β-catenin, WNT, cell cycle arrest, migration

## Abstract

*Background and Objectives*: PNU-74654, a Wnt/β-catenin pathway inhibitor, has an antiproliferative effect on many cancer types; however, its therapeutic role in pancreatic cancer (PC) has not yet been demonstrated. Here, the effects of PNU-74654 on proliferation and cell cycle phase distribution were studied in PC cell lines. *Materials and Methods*: The cancer-related molecular pathways regulated by PNU-74654 were determined by a proteome profiling oncology array and confirmed by western blotting. *Results*: The cell viability and proliferative ability of PC cells were decreased by PNU-74654 treatment. G1 arrest was observed, as indicated by the downregulation of cyclin E and cyclin-dependent kinase 2 (CDK2) and the upregulation of p27. PNU-74654 inhibited the epithelial–mesenchymal transition (EMT), as determined by an increase in E-cadherin and decreases in N-cadherin, ZEB1, and hypoxia-inducible factor-1 alpha (HIF-1α). PNU-74654 also suppressed cytoplasmic and nuclear β-catenin and impaired the NF-κB pathway. *Conclusions*: These results demonstrate that PNU-74654 modulates G1/S regulatory proteins and inhibits the EMT, thereby suppressing PC cell proliferation, migration, and invasion. The synergistic effect of PNU-74654 and chemotherapy or the exclusive use of PNU-74654 may be therapeutic options for PC and require further investigation.

## 1. Introduction

Pancreatic cancer (PC), a lethal disease, responds poorly to conventional cancer treatments. Although PC accounts for 2.6% of new cancer cases, it causes 466,003 new deaths and ranks 7th in terms of cancer deaths worldwide [1]. The early detection of asymptomatic PC also needs further improvement because of the current lack of adequate screening methods [2]. PC has the lowest five-year relative survival rate in the world due to the late-stage diagnosis that occurs in more than 80% of PC cases [2,3]. Even in developed countries, the current PC treatments have only limited effects, with high mortality rates [4]. The main type of PC tumor is adenocarcinoma, accounting for nearly 85% of cases, while pancreatic neuroendocrine tumors account for less than 5% [5,6]. In patients with resectable PC, the overall survival (OS) and 5-year survival are only 28 months and 28.8%, respectively, with a combination of gemcitabine and capecitabine being the standard adjuvant therapy after surgery [7]. For patients with unresectable PC, gemcitabine has been used for years as a standard treatment, but it has a 1-year survival rate of merely 19% and a median progression-free survival (PFS) of only 115 days [8]. Among all the combination chemotherapies, FOLFIRINOX (oxaliplatin, irinotecan, leucovorin, and fluorouracil) is reported to have a better OS and median PFS than gemcitabine alone [9]. The association between current chemotherapies and the poor response and the lowest survival rate among solid tumors clearly indicates the need for a new regimen.

Several cancers, including PC, colorectal cancer, and hepatocellular carcinoma, are triggered by activation mutations of the Wnt/β-catenin pathway [10,11,12]. The Wnt/β-catenin-dependent pathway regulates the accumulation of the transcriptional coactivator β-catenin in the nucleus after Wnt ligand binding to LRP-5/6 receptors [13]. The accumulation of β-catenin in the nucleus activates several Wnt target genes, such as VEGF, cyclin D1, c-Myc, and MMP7, through the interaction between β-catenin and the T cell-specific factor (TCF)/lymphoid enhancer binding factor (LEF) to cause angiogenesis and cell cycle progression and to inhibit apoptosis [14]. The Wnt/β-catenin-dependent pathway therefore plays an important role in cell regulation. In PC, the activation of Wnt/β-catenin is correlated with the expression of VEGF and a consequent increase in the number of blood vessels, larger tumor size, and poor prognosis [12,15]. Previous studies have shown an association between cyclin D1 overexpression and the accumulation of β-catenin. For example, cancer patients with high cyclin D1 overexpression had poorer median survival [16,17]. Elevated β-catenin expression is also correlated with the evasion of apoptosis and increased chemoresistance in PC due to the upregulation of survivin, the inhibition of tumor apoptosis, and the promotion of resistance to gemcitabine regimens [18,19,20]. These observations suggest that the suppression of the Wnt/β-catenin pathway could be an effective treatment strategy for PC.

**Figure 1:**
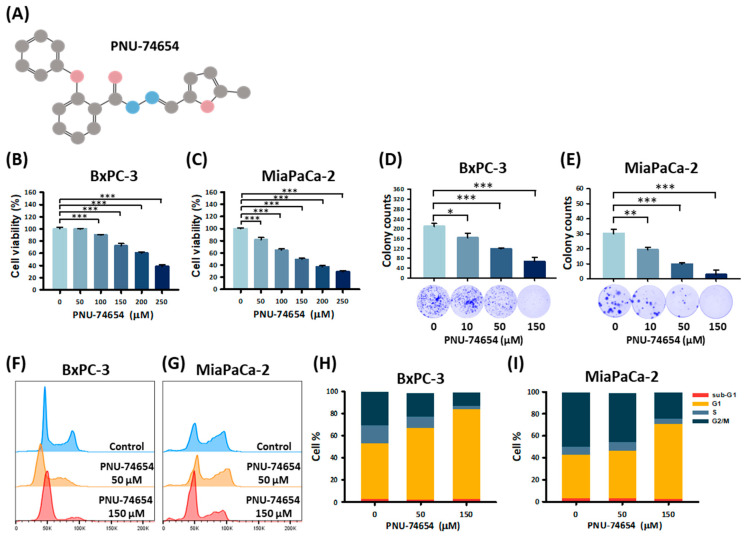
PNU-74654 inhibited cell viability and clonogenic activity and promoted G1 arrest. (**A**) Chemical structure of PNU-74654. IUPAC name: 2-Phenoxybenzoic acid-[(5-methyl-2-furanyl)methylene]hydrazide. Formula: C_19_H_16_N_2_O_3_. SMILES: CC1=CC=C(O1)C=NNC(=O)C2=CC=CC=C2OC3=CC=CC=C3. (**B**,**C**) Cell viability was determined using the MTT assay after treating BxPC-3 and MiaPaCa-2 cells with the indicated concentrations of PNU-74654 for 24 h. PNU-74654 treatment resulted in a dose-dependent reduction in pancreatic cancer cell viability. (**D**,**E**) Colony formation assays were executed to examine cell growth changes in response to treatment with PNU-74654 (0, 10, 50, and 150 μM) for 10 days. PNU-74654 inhibited the proliferation ability of both cell lines. (**F**,**G**) The cell cycle was analyzed using PI staining and flow cytometry. (**H**,**I**) Quantification of the PI staining of BxPC-3 and MiaPaCa-2 cells. PNU-74654 induced G1 cell cycle arrest in both cell lines. Bars depict the mean ± SD. * *p* < 0.05, ** *p* < 0.01, and *** *p* < 0.001.

One known Wnt/β-catenin pathway inhibitor is PNU-74654 (Figure 1A), a compound that shows an anticarcinogenic response against several types of cancer [21,22]. PNU-74654 has a molecular weight of 320.34; it is soluble in ethanol (10 mg/mL (31.22 mM)) but insoluble in water. In NCI-H295 cells, PNU-74654 was shown to function by physically binding to β-catenin to prevent the binding of β-catenin and T cell factor 4 (Tcf4), thereby reducing cell proliferation, promoting apoptosis, inhibiting the accumulation of β-catenin in the nucleus, and suppressing CTNNB1/β-catenin expression [21,22,23]. Growing evidence now indicates that the antitumor activity of PNU-74654 plays an important role in some cancers. For example, PNU-74654 can inhibit the migration and invasion of breast cancer (BC) cells by upregulating E-cadherin and downregulating MMP3 and MMP9 to synergistically enhance the anticancer effect of fluorouracil (5-FU) [22]. The same synergistic effect was also observed for colorectal cancer, as the combination of PNU-74654 plus 5-FU increased the levels of reactive oxygen species in CT-26 cells, thereby promoting the sensitivity of cancer cells to chemotherapy drugs [24]. The antiproliferative and antimigration effects of PNU-74654 against hepatocellular carcinoma have also been demonstrated, as this inhibitor suppressed the nuclear factor κB (NF-κB) pathway and disrupted the cell cycle of cancer cells [25]. However, despite many preclinical studies, the mechanism underlying the anticancer effects of PNU-74654 in PC is not yet clear. The aim of the present study was to investigate the antiproliferative activity of PNU-74654 and its effects on the expression of Wnt pathway-related proteins in PC cell lines to clarify the anticancer role of PNU-74654.

## 2. Materials and Methods

### 2.1. Cell Culture

The PC cell lines BxPC-3 and MiaPaCa-2 were obtained from the Japanese Collection of Research Bioresources Cell Bank (JCRB, Ibaraki, Japan), cultured, and stored based on the suppliers’ instructions. The cells were maintained in RPMI-1640 medium supplemented with 10% fetal bovine serum (FBS), 100 U/mL penicillin, 100 μg/mL streptomycin, 2 g/mL NaHCO_3_, 1 mM sodium pyruvate, and 0.1 mM NEAA. All procedures were performed as described previously [26,27,28,29].

### 2.2. MTT Assay

The MTT assay was used to evaluate cell viability and drug cytotoxicity. BxPC-3 and MiaPaCa-2 cells were seeded in triplicate at a density of 1 × 10^4^ cells per well in 96-well plates, allowed to attach overnight, and then treated with PNU-74654 (0, 50, 100, 150, 200, or 250 μM; MedChemExpress, Monmouth Junction, NJ, USA) for 24 h. PNU-74654 was dissolved in dimethyl sulfoxide. The MTT solution (0.5 mg/mL) was added to each well and incubated. The formazan product was dissolved in 100 μL DMSO, and the absorbance was measured with an ELISA reader at 570 nm, as described previously [28,29].

### 2.3. Colony Formation Assay

BxPC-3 (500 cells) and MiaPaCa-2 (250 cells) were seeded in triplicate as single cells in a 6-well plate for 10 days. The cells were then treated with PNU-74654 (0, 10, 50, or 150 μM). The resulting colonies were fixed with 95% ethanol and stained with 20% Giemsa solution, as described previously [26,28]. Each experiment was performed in triplicate in three independent experiments.

### 2.4. Cell Cycle Analysis

The cell cycle of the BxPC-3 and MiaPaCa-2 cells was analyzed by flow cytometry (FACSCanto™ II Cell Analyzer; BD Biosciences, Franklin Lakes, NJ, USA), as described previously [27,28,29]. BxPC-3 and MiaPaCa-2 cells were plated in 6-well plates (4.0 × 10^5^ and 3.0 × 10^5^ cells, respectively) and treated with PNU-74654 (50 μM or 150 μM) for 24 h. For the cell cycle phase distribution, BxPC-3 and MiaPaCa-2 cells were fixed in 70% (*v*/*v*) cold ethanol. Phosphate-buffered saline (PBS) (containing 0.4 µg/mL propidium iodide and 0.5 mg/mL RNase) was used to resuspend the fixed cells. Cell cycle phase distribution was analyzed using FlowJo software version 10 (BD Biosciences, Franklin Lakes, NJ, USA).

### 2.5. Wound-Healing Assay

An Ibidi culture insert (Ibidi, Munich, Germany) was used to perform the wound-healing assay. We seeded BxPC-3 and MiaPaCa-2 cells on both sides of an Ibidi culture insert (Ibidi, Gräfelfing, Germany) at a density of 3.0 × 10^4^ cells per well, cultured overnight, and then removed the insert. The cells were treated with PNU-74654 (0, 50, or 100 μM) in culture medium containing 0.5% fetal bovine serum (FBS) and incubated for 36 or 48 h. Images were photographed at each time point (0, 24, and 36 h or 0, 24, and 48 h) and analyzed using ImageJ software version 1.52a (National Institutes of Health, Bethesda, MD, USA). Each experiment was performed in triplicate, and the cell migration number or area of each group was calculated.

### 2.6. Transwell Invasion Assay

Triplicate Transwell (Cat# 3422, Corning, NY, USA) assays were used to analyze cell invasion. BxPC-3 and MiaPaCa-2 cells were suspended in serum-free medium containing PNU-74654 (50 and 100 μM). A 0.2% Matrigel (Millipore, Darmstadt, Germany) mixture diluted in FBS-containing culture medium was applied to cover the upper side of the filter. The cells were then allowed to invade for 24 h. The cells that adhered to the underside of the Transwell membrane were fixed with 95% ethanol and stained with 1% crystal violet solution. The cells on the upper side of the membrane were removed using wet cotton swabs. The number of invading cells per mm^2^ was counted for each group using TissueFAX Plus software (Version 2.0, Vienna, Austria).

### 2.7. Western Blotting

BxPC-3 and MiaPaCa-2 cells cultured in PNU-74654 (0, 50, and 150 μM) for 24 h were harvested with 100 μL PNU-74654 or RIPA lysis buffer containing a protease inhibitor cocktail (Roche Applied Science, Penzberg, Germany). The lysed cells were scraped into Eppendorf tubes and collected by centrifugation (10,400 rpm for 20 min at 4 °C). The supernatant was stored at −80 °C. Total protein (30 μg) in the supernatant was separated by 6–15% SDS-PAGE and electroblotted onto a polyvinylidene fluoride membrane, which was then blocked (5% nonfat dry milk) for 2 h. The membranes were probed and incubated at 4 °C overnight with an appropriate antibody (dilution: 1/1000): Rb (A3618, ABclonal), phosphate retinoblastoma protein (p-Rb) (AP0117, ABclonal, Woburn, MA, USA), cyclin E (A14225, ABclonal), cyclin-dependent kinase 2 (CDK2) (A18000, ABclonal), p21 (10355-1-AP, Proteintech, Rosemont, IL, USA), p27 (A19095, ABclonal), actin (AC026, ABclonal), hypoxia-inducible factor-1 alpha (HIF-1α) (610958, BD, Franklin Lakes, NJ, USA), β-catenin (A19657, ABclonal), E-cadherin (3195, CST, Danvers, MA, USA), N-cadherin (22019-1-AP, Proteintech), claudin-1 (13995, CST), and ZEB1 (70512, CST). The membranes were washed with Tris-buffered saline containing Tween 20 (TBST). and then incubated with secondary antibodies at room temperature for 1 h. After a further wash with TBST, the membranes were reacted with Immobilon™ Western Chemiluminescent HRP Substrate (Merck Millipore, Burlington, MA, USA) and visualized using a GE Healthcare ImageQuant LAS4000 instrument (GE Healthcare, Marlborough, MA, USA). The images were quantified with AlphaEase FC software version 6.0 (Alpha Innotech Corporation, San Leandro, CA, USA). All procedures were conducted as described previously [28,29].

### 2.8. Isolation of Nuclear and Cytoplasmic Proteins

The nuclear and cytoplasmic fractions from the BxPC-3 and MiaPaCa-2 cells were isolated using Cayman’s Nuclear Extraction Kit (Ann Arbor, MI, USA). The nuclear and cytoplasmic extracts were purified using the following steps: BxPC-3 and MiaPaCa-2 cells were collected and centrifuged (300× *g* for 5 min at 4 °C). The supernatant was removed and the cell pellet was resuspended in PBS/phosphatase inhibitor solution and centrifuged (300× *g* for 5 min at 4 °C); the resuspension and centrifugation steps were repeated twice. The cell pellet was then resuspended in complete hypotonic buffer and incubated on ice for 15 min. NP-40 assay reagent was added and the cells were centrifuged (14,000× *g*) for 30 s at 4 °C. The supernatant containing the cytoplasmic fractions was collected and stored. The remaining pellet was resuspended in complete nuclear extraction buffer and centrifuged (14,000× *g* for 10 min at 4 °C). The supernatant containing the nuclear fractions was collected and stored.

### 2.9. Proteome Profiling with a Human XL Oncology Array

Cancer-related protein expression was measured using the Proteome Profiler Human XL Oncology Array Kit (R&D Systems, Minneapolis, MN, USA), as previously described [29]. The BxPC-3 and MiaPaCa-2 cells were treated with PNU-74654 (150 μM) for 24 h and then collected and lysed for analysis. A 200 μg sample of total protein was incubated overnight with the nitrocellulose membranes of the oncology array to determine the relative expression levels of 84 human cancer-related proteins between samples. After washing to remove the unbound antibodies, the membranes were incubated in a detection antibody cocktail for 1 h, followed by the addition of a chemiluminescent detection reagent. The cells were examined using an ImageQuant LAS4000 instrument (GE Healthcare, Marlborough, MA, USA). ImageJ software version 1.52a (National Institutes of Health, Bethesda, MD, USA) was used for quantification.

### 2.10. Statistical Analysis

We used IBM SPSS software v.20.0 (Armonk, NY, USA) for statistical analysis. Data are shown as the mean ± standard deviation. Student’s *t*-tests were performed to determine differences between groups, and all tests were two-sided. A *p* value of <0.05 was considered statistically significant.

## 3. Results

### 3.1. PNU-74654 Suppresses PC Cell Proliferation by Promoting Cell Cycle Arrest

Figure 1A shows the chemical structure of PNU-74654. PNU-74654 dose-dependently reduced PC cell viability at the tested concentrations (Figure 1B,C). The proliferation ability of both cell lines showed a dose-dependent decline, as indicated by the colony formation assays (Figure 1D,E). When treated with 150 μM PNU-74654, the proportion of cells in the G1 phase in both cell lines was significantly higher in the treated cells than in the control cells (30.7% and 28.4%, *p* = 0.02 and *p* = 0.02, respectively; Figure 1F–I). The proportion of BxPC-3 cells in the G1 phase was also significantly increased after treatment with 50 μM PNU-74654 (14.4%; *p* = 0.02). However, the percentage of apoptotic cells remained unchanged, regardless of treatment. Treatment with PNU-74654 (0, 50, or 150 μM) resulted in percentages of apoptotic cells of 3.2 ± 0.8, 2.9 ± 0.9, and 1.6 ± 0.5, respectively, for BxPC-3 cells and 5.6 ± 0.4, 5.8 ± 0.5, and 8.0 ± 0.4, respectively, for MiaPaCa-2 cells.

### 3.2. PNU-74654 Inhibits the Migration of PC Cells

**Figure 2:**
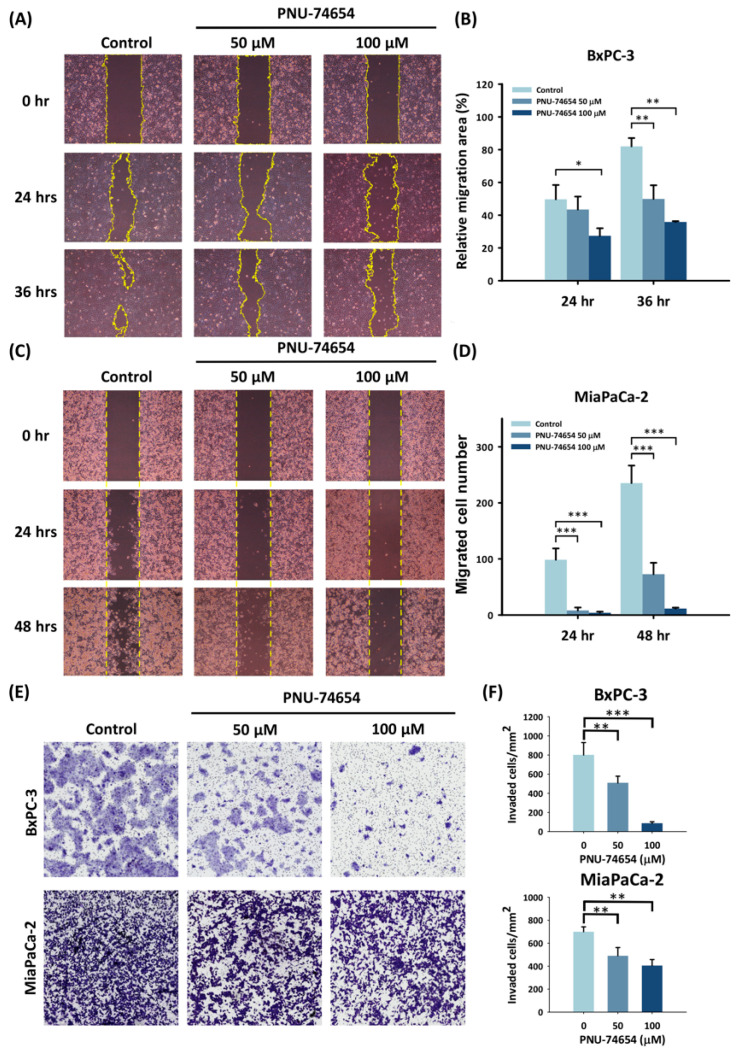
PNU-74654 reduced pancreatic cancer cell migration. (**A**,**C**) Representative figures of cell migration were analyzed by the wound-healing assay and (**B**,**D**) quantified in BxPC-3 and MiaPaCa-2 cells. The migration area of the BxPC-3 cells and the number of migrated MiaPaCa-2 cells decreased following treatment with PNU-74654. (**E**) The invasion ability of BxPC-3 and MiaPaCa-2 cells was evaluated by Transwell assays and (**F**) quantified. PNU-74654 treatment reduced the invading cell numbers. Bars depict the mean ± SD. * *p* < 0.05, ** *p* < 0.01, and *** *p* < 0.001.

Migration ability is a major characteristic of malignant cancers. The wound-healing assay identified a significantly smaller migration area for BxPC-3 cells treated with 100 μM PNU-74654 for either 24 or 36 h than for control cells (32.1% and 46.2%, *p* = 0.006 and *p* = 0.004, respectively; Figure 2A,B). The MiaPaCa-2 cells had significantly smaller numbers of migrated cells per mm^2^ for cells treated with 100 μM PNU-74654 for 24 and 48 h (4.00 and 11.5, *p* = 0.01 and *p* = 0.01, respectively; Figure 2C,D) than for control cells (98.50 and 235.33, respectively).

### 3.3. PC Cell Invasion Is Suppressed by PNU-74654

The Transwell invasion assays showed a significant reduction in the invasive cell counts for both the BxPC-3 and MiaPaCa-2 cell lines (*p* < 0.05) (Figure 2E,F). PNU-74654 treatment significantly and dose-dependently inhibited the invasive behavior of both cell lines.

### 3.4. Molecular Mechanism of PNU-74654-Induced Cell Cycle Arrest and Inhibition of the Epithelial–Mesenchymal Transition

**Figure 3:**
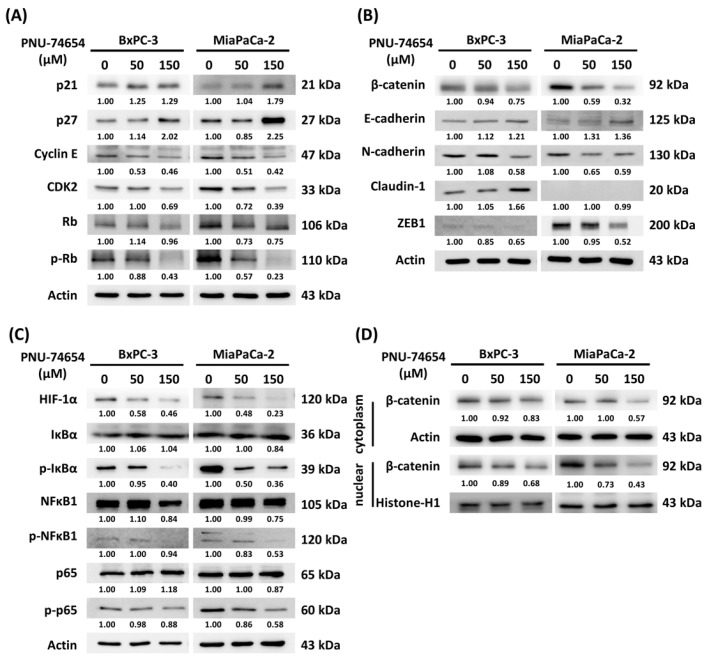
PNU-74654 induced cell cycle arrest and inhibited the acquisition of the mesenchymal phenotype. Western blots of BxPC-3 and MiaPaCa-2 cells showed expression of (**A**) cell cycle-associated proteins, (**B**) EMT markers, (**C**) HIF-1α and NF-κB-associated proteins, and (**D**) cytoplasmic and nuclear β-catenin. PNU-74654 altered the expression of cell cycle-related proteins and promoted the epithelial phenotype of pancreatic cancer cells, which caused cell cycle arrest and EMT inhibition.

Consistent with the results of the flow cytometry study in Figure 1F,G, PNU-74654 treatment (50 and 150 μM for 24 h) induced G1 cell cycle arrest, as indicated by the downregulation of cyclin E and CDK2, which control cell cycle progression, and promotion of the CDK inhibitor p27 (Figure 3A). The expression of p-RB, a protein associated with the transcription of cell cycle genes, was also suppressed by PNU-74654 treatment.

The EMT-associated proteins were also evaluated because of their involvement in the inhibition of cell migration. Figure 3B shows the enhancement of the expression of E-cadherin, a molecule associated with an epithelial phenotype in the PNU-74654-treated BxPC-3 and MiaPaCa-2 cells, but the suppression of the expression of mesenchymal phenotype markers (N-cadherin and ZEB1). β-Catenin, which can interact with E-cadherin to enhance cell–cell adhesion, was also downregulated by PNU-74654 treatment. By contrast, the expression of HIF-1α, a promoter of hypoxia-induced EMT, was inhibited by PNU-74654 treatment (Figure 3C). The downregulation of HIF-1α was also evident in the proteome profile experiment.

The NF-κB pathway was also inhibited by PNU-74654 treatment. The activity of the NF-κB family members, including NF-κB1 and p65, was elevated following their phosphorylation, but PNU-74654 treatment suppressed p-NF-κB1 and p-p65 expression. The phosphorylation of IκB, a NF-κB inhibitor, leads to its degradation and NF-κB activation, and p-IκB expression was suppressed by PNU-74654 treatment.

Wnt target genes were activated once β-catenin protein accumulated in the nucleus. The isolation of cytosolic and nuclear β-catenin confirmed the downregulation of cytoplasmic and nuclear β-catenin in both cell lines after PNU-74654 treatment (Figure 3D). PNU-74654 altered the expression of cell cycle-related proteins and promoted the epithelial phenotype of PC cells, resulting in cell cycle G1 arrest and the suppression of tumor cell motility.

## 4. Discussion

This is the first study to report an antiproliferative role for PNU-74654, a Wnt/β-catenin pathway inhibitor, in PC. The exclusive in vitro use of PNU-74654 in cancer cells, especially in PC cells, has not been investigated in previous studies. PNU-74654 induced cell cycle arrest by downregulating the expression of p27, which is normally promoted by cyclin E–CDK2 and by suppressing the expression of p-RB, resulting in the inhibition of cancer growth. The migration and invasion abilities of PC cells were decreased by PNU-74654 treatment, which also suppressed the EMT, as confirmed by increased expression of E-cadherin and decreased expression of N-cadherin and ZEB1. The expression of HIF-1α, a promoter of hypoxia-induced EMT, was also downregulated, leading to a decrease in both cytoplasmic and nuclear β-catenin. PNU-74654 treatment also inhibited the NF-κB pathway, which could potently suppress the proliferative properties of cancer cells. These findings confirmed the anticancer effect of PNU-74654 and suggested a possible mechanism.

The Wnt/β-catenin pathway is a crucial signal transduction pathway that participates in many biological functions, including cell proliferation, tissue homeostasis, and carcinogenesis [30,31]. β-Catenin is a key player in the induction of Wnt signaling and is known to aid in stabilizing cell–cell adhesive junctions and regulate transcription [30]. The multiprotein complex, which consists of adenomatous polyposis coli (APC), axin, and glycogen synthase kinase 3β (GSK3β), can bind to β-catenin and regulate its degradation. The dissociation of the APC/Axin/GSK3β complex is triggered by the binding of the Wnt ligand to the Frizzled cell surface receptor, which leads to the cellular accumulation of β-catenin and the entry of β-catenin into the nucleus [32,33]. The β-catenin that accumulates in the nucleus interacts with TCF/LEF and activates the expression of target genes, including c-Myc, VEGF, and cyclin D1, to promote the genesis of cancers [14]. In general, the results presented here suggest that Wnt/β-catenin inhibitors may have important anticancer effects.

Hypoxia-inducible factor 1 α (HIF-1α) is an important factor by which mammalian cells adapt to hypoxic conditions [34,35]. Under normoxia, prolyl residues in the oxygen-dependent degradation domain (ODDD) of HIF-α are hydroxylated by prolyl hydroxylase domain-containing (PHD) proteins. Hydroxylated HIF-1α then interacts with von Hippel–Lindau (pVHL) and undergoes ubiquitination and degradation. Under hypoxic conditions, HIF-1α degradation is suppressed due to the inhibition of prolyl hydroxylation, thereby minimizing the effects of oxygen deprivation. The accumulation of HIF-1α alters cellular metabolism, induces angiogenesis, and regulates the signaling pathways involved in cell proliferation and survival [34,36,37]. These responses play crucial roles in the progression of cancers, including pancreatic cancer [34,36,37]. Hence, HIF-1α signaling regulation may be a promising strategy for decreasing the malignancy of pancreatic cancer.

The genes targeted by NF-κB play significant roles in cell survival, proliferation, inflammatory responses, and angiogenesis [38]. Once activated, NF-κB can initiate the transcription of several genes and regulate cancer progression [39,40]. The inhibition of the NF-κB pathway suppresses the growth of tumors, including pancreatic cancers [40]. The activation of NF-κB is primarily triggered by the site-specific phosphorylation of inhibitory molecules, such as IκBα, through the action of IκB kinase (IKK) [41]. The two N-terminal serines of IκBα are phosphorylated, inducing the degradation of IκBα in the proteasome [38]. In addition to the phosphorylation and subsequent degradation of inhibitory molecules, protein kinases are also essential for the effective activation of NF-κB family members, including the two main members, NF-κB1 and p65, by phosphorylating the functional domains of the NF-κB proteins themselves [42,43]. Our findings indicate that inhibition using PNU-74654 can be a potent anticancer strategy, as treatment with this inhibitor suppresses the expression of p-IκB, p-NF-κB1, and p-p65.

PNU-74654 suppresses the carcinogenesis of several types of cancer. For example, in breast cancer (BC), PNU-74654 can inhibit cell migration and invasion by upregulating E-cadherin and downregulating MMP3 and MMP9, thereby providing a synergistic enhancement of the anticancer effect of fluorouracil (5-FU) [22]. The same synergistic effect was also reported for colorectal cancer [24]. The findings presented here confirmed that the inhibition of cyclin D1 and survivin by PNU-74654 reduced cancer cell growth. Furthermore, tumor growth was inhibited by the combined use of PNU-74654 and 5-FU by altering the expression of reactive oxygen species, superoxide dismutase, MCP-1, p53, and TNF-α. In hepatocellular carcinoma, the PNU-74654 treatment of HepG2 and Huh7 cells suppressed NF-κB pathway activity, thereby interfering with the cell cycle and promoting antiproliferative and antimigration effects [25]. In adrenocortical cancer, PNU-74654 treatment prevented the binding of TCF to β-catenin and decreased CTNNB1 mRNA expression and nuclear β-catenin accumulation, thereby promoting apoptosis cells, reducing cell viability, and impairing adrenal steroid secretion [21].

Our flow cytometry data revealed a significant increase in the numbers of BxPC-3 and MiaPaCa-2 cells in the G1 phase, indicating G1 arrest and growth inhibition. The viability and proliferative ability of PC cells were dose-dependently inhibited by PNU-74654 treatment, in agreement with previous findings for BC [22]. Further investigation of the BxPC-3 and MiaPaCa-2 cells revealed that PNU-74564, as a Wnt/β-catenin pathway inhibitor, reduced both cytoplasmic and nuclear β-catenin expression. In addition, cyclin E-CDK2, the regulator of G1–S phase progression, was downregulated by PNU-74654 treatment, whereas p27, a cyclin-dependent kinase inhibitor, was upregulated. Moreover, PNU-74654 suppressed p-RB, which is a response to cell cycle gene transcription. The expression of epithelial phenotype-associated molecules (e.g., E-cadherin) was increased, as also reported for BC [22], while the expression of mesenchymal phenotype-associated molecules (e.g., N-cadherin and ZEB1) was suppressed. The expression of HIF-1α and the NF-κB pathway was also suppressed in PC after treatment with PNU-74654. The western blotting data confirmed the antiproliferative effect of PNU-74654 on PC.

Current studies focusing on the Wnt/β-catenin pathway may confirm our results. Many Wnt/β-catenin inhibitors are presently undergoing clinical trials as treatments for numerous cancer types. LGK974 can selectively inhibit porcupine, which controls Wnt secretion and suppresses tumor proliferation [44]. Cell cycle arrest can be induced by LGK974 by downregulating cyclin D1, c-Myc, MMP9, and MMP2 in renal cancer cells [45]. The anticancer efficacy of LGK974 for *NOTCH*-deficient head and neck squamous cell carcinoma has also been reported [46]. The interaction between β-catenin and the CREB-binding protein (β-catenin transcriptional coactivator) can be suppressed by PRI-724, a specific CBP/catenin antagonist [14,47]. PRI-724 significantly increased the percentage of hepatocellular carcinoma cells in the G0/G1 cell cycle phases and induced G0/G1 arrest, along with an increase in p21 Waf1/Cip1 expression and a reduction in c-Myc and Skp2 expression [48].

The Wnt/β-catenin pathway also plays an important role in the EMT, suggesting that inhibition of this pathway inhibitor may reduce cancer malignancy and potentially inhibit tumor metastasis. Some studies have shown that IWR-1, a new WNT/β-catenin pathway inhibitor, suppresses survivin expression and inhibits the EMT in colorectal cancer cells [49]. Another Wnt signaling inhibitor, XAV939, when administered in combination with paclitaxel, can also cause EMT inhibition by upregulating E-cadherin expression in BC [50].

This study has some limitations to consider. One is that the BxPC-3 and MiaPaCa-2 cell lines are not fully representative of all types of PC. In addition, the study lacked a positive control, such as a HIF-1alpha inhibitor or a non-specific EMT inhibitor. Furthermore, various treatment dosages were utilized in this study. Furthermore, a dosage of 250 µM did not cause significant pancreatic cancer cell death. Since higher dosages did not provide any additional benefits, we opted against employing high-dose treatments. When examining colony formation over an extended period after administering PNU-74654, we also noticed a limited number of colonies at the 150 µM dose. Consequently, we increased the dosage by 10 µM to validate the observed trend. Due to the considerable impact of 150 µM PNU-74654 on the cell cycle, we did not assess its effects on migration and invasion. Another limitation is that we only evaluated the mechanisms of the antiproliferative effects by western blotting; therefore, overexpression models need further testing to verify their validity. This was also an in vitro study and was conducted under normoxic conditions. The effects of PNU-74654 under low oxygen and in vivo conditions also warrant investigation. Finally, for clinical use, the toxicity of PNU-74654 treatment must also be considered.

## 5. Conclusions

Our findings support that PNU-74654, as a WNT/β-catenin pathway inhibitor, reduced the viability and proliferative ability of PC. PNU-74654 modulated the G1–S regulator protein and inhibited the EMT marker, resulting in cell cycle arrest and the inhibition of cancer proliferation, migration, and invasion. These findings suggest novel therapeutic insights into the treatment of PC. The synergistic effect of PNU-74654 and chemotherapy, or the exclusive use of PNU-74654, on PC may be a therapeutic choice and require further investigation.

## Data Availability

The datasets used and/or analyzed during the current study are available from the corresponding author upon reasonable request.
